# Knowledge, Attitudes, and Willingness of Healthcare Workers in Iraq’s Kurdistan Region to Vaccinate against Human Monkeypox: A Nationwide Cross-Sectional Study

**DOI:** 10.3390/vaccines11121734

**Published:** 2023-11-21

**Authors:** Sirwan Khalid Ahmed, Salar Omer Abdulqadir, Rukhsar Muhammad Omar, Safin Hussein, Karzan Qurbani, Mona Gamal Mohamed, Hazhar Talaat Abubaker Blbas, Mathumalar Loganathan Fahrni, Antonio Ivan Lazzarino

**Affiliations:** 1 Department of Adult Nursing, College of Nursing, University of Raparin, Rania, Sulaymaniyah 46012, Iraq; sirwan.k.ahmed@gmail.com; 2 Ministry of Health, General Directorate of Health-Raparin, Rania, Sulaymaniyah 46012, Iraq; 3 Department of Psychiatric and Mental Health Nursing, College of Nursing, University of Raparin, Rania, Sulaymaniyah 46012, Iraq; 4 Department of Kindergarten, College of Basic Education, University of Raparin, Rania, Sulaymaniyah 46012, Iraq; 5 Department of Biology, College of Science, University of Raparin, Rania, Sulaymaniyah 46012, Iraq; 6 Department of Adult Nursing, RAK College of Nursing, RAK Medical and Health Sciences University, Ras Al Khaimah 72603, United Arab Emirates; 7 Department of Statistics, College of Administration and Economics, Salahaddin University, Erbil 44001, Iraq; 8 Faculty of Pharmacy, Universiti Teknologi MARA (UiTM), Puncak Alam Campus, Selangor Branch, Puncak Alam 42300, Malaysia; 9 Department of Epidemiology and Biostatistics, Imperial College London, Norfolk Place, London W2 1PG, UK

**Keywords:** monkeypox virus, monkeypox, knowledge, attitude, vaccine acceptance, vaccine hesitancy, healthcare workers, doctors, nurses, pharmacists

## Abstract

Although human monkeypox infections had not been recorded in the Kurdistan region of Iraq as of August 2023, the rapid growth of cases worldwide and the detection of monkeypox in neighboring Middle Eastern nations call for careful planning and timely response measures. Educating and empowering frontline healthcare workers (HCWs) so that they can act to curb the spread of monkeypox infections are core elements of primary prevention and protecting public health. Therefore, this study aimed to assess HCWs’ knowledge and attitudes about monkeypox and their willingness to vaccinate against monkeypox. By employing a convenience sampling method, an online survey was disseminated via Google Forms between 1 November 2022 and 15 January 2023. The researchers utilized regression analyses to ascertain the factors associated with the three parameters: knowledge, attitude, and the willingness to vaccinate. A total of 637 HCWs were included in the analysis (ages ranged between 21 and 51 years). The mean overall scores were 8.18 of a max score of 16 (SD 3.37), 3.4 of 5 (SD 1.37), and 2.41 of 5 (SD 1.25) for knowledge, attitude, and willingness to vaccinate, respectively. A multivariate logistic regression analysis demonstrated that HCWs who had heard about monkeypox before 2022 rather than later had a higher level of knowledge (AOR: 4.85; 95% CI: 2.81–8.36; *p* < 0.001). In addition, those who had newly joined the workforce or had less than 1 year experience in practice had more positive attitudes about curbing monkeypox (AOR: 0.35; 95% CI: 0.20–0.59; *p* < 0.01) than those who practiced for longer. No significant predictors of willingness to vaccinate against monkeypox were identified. The research revealed that HCWs exhibited a relatively low level of monkeypox knowledge. They also had poor attitudes towards monkeypox vaccination and were therefore reluctant to receive the vaccines. Imparting knowledge about the infectious disease can cultivate better awareness and attitudes among HCWs as to their roles in mitigating the spread of an epidemic in the foreseeable future.

## 1. Introduction

While the world was still coping with the aftermath of the COVID-19 pandemic, it was hit with another wave of an infectious disease, spreading, and threatening to become a pandemic. The monkeypox outbreak holds significance in the context of the ongoing COVID-19 epidemic, as a new outbreak can further strain healthcare systems already under pressure and on the verge of collapse. The emergence of a new infectious disease adds complexity to the global health crisis, requiring additional resources and attention. In 1958, researchers in Denmark discovered the first incidence of monkeypox virus (MPXV) in a group of monkeys [1]. Following that, in 1970, the MPXV, an orthopox DNA virus that is zoonotic, was first discovered in humans in the Democratic Republic of the Congo (formerly Zaire) [2]. Subsequently, the disease became endemic throughout the West and Central Africa [3,4,5]. Since then, in 2003, the first case of a monkeypox infection was recorded in non-endemic nations, caused by an imported rodent from Ghana [3,4,6,7]. Monkeypox cases were then recorded in the United Kingdom, Israel, and Singapore between 2018 and 2019 [8,9,10]. In May 2022, a significant number of cases were reported from nations with no known history of monkeypox transmission [11]. On 23 July, 2022, the World Health Organization (WHO) declared the monkeypox outbreak as a public health emergency of international concern (PHEIC) [12]. As of 5 July 2023, there were 88,122 cases confirmed by laboratory testing, with 148 deaths reported across 112 countries [13]. The monkeypox outbreak that occurred in 2022 was sporadic and had varying characteristics in comparison to its prior presentations such as a prolonged incubation period (up to 21 days), occurrence of infections outside an endemic region, a high prevalence in males, sexual transmission, anogenital lesions, and the involvement of younger patients [1,2,14,15,16,17,18,19,20].

Humans can contract monkeypox by direct contact with infected animals, humans, and contaminated surfaces and raw meat [21,22,23,24]. Furthermore, it is possible that squirrels, prairie dogs, and rodents contributed to the spread of MPXV to humans [22,25]. The recent discovery of MPXV transmission from humans to dogs underscores the importance of researching the dynamics of monkeypox dissemination in depth [26,27]. Although humans in close contact with infected cases are susceptible to contracting the virus, the present trend appears to be heavily focused on men who engage in sexual activity with other men (MSM) [2,18,28]. Incidences have also been observed in females and children [29,30,31,32]. Notwithstanding, an overwhelming number of reported cases of monkeypox in recent studies conducted in the UK and Spain were found to be among MSM [14,18]. As the MSM community is disproportionately affected by the monkeypox virus, close attention must be paid to the problems of stigmatization and discrimination, which can be just as devastating as contracting the virus [33,34,35]. Until today, little is known about the potential risk factors for transmission and infection, zoonotic hosts, and vectors [17,36]. A number of circumstances, including close contact between humans and infected animals, the cessation of smallpox vaccination, and increasing international travel, can render the disease a global public health threat in the future [7,37].

Monkeypox is a self-limiting disease with a case fatality rate between 1% and 10% [38]. There were only a very low number of fatalities reported during the ongoing outbreak [39]. Symptoms included fever, headache, back pain, myalgia, fatigue, lymphadenopathy, and a variety of skin lesions (including papules, pustules, and ulcers) on the face and body [11,40,41]. The progression of this skin eruption includes the appearance of macules, papules, pustules, vesicles, and finally scabs [42]. Lymphadenopathy appears to be one of the primary features distinguishing monkeypox from smallpox [37]. There are numerous complications associated with monkeypox, such as keratitis, paraphimosis, encephalitis, pneumonitis, myocarditis, conjunctivitis, and secondary bacterial infections [2,43,44,45,46,47,48,49]. Children, pregnant woman, and immunosuppressed individuals, particularly HIV-positive people, are at elevated risks for severe outcomes [50,51,52,53], but whether or not antiretroviral treatment (ART) is effective in reducing the risks is uncertain [54].

There is currently no MPXV-specific vaccine or drugs available for use. Nevertheless, antiviral medications such as Cidofovir, Tecovirimat (TPOXX), Brincidofovir, and Vaccinia Immune Globulin Intravenous (VIGIV) have proven to be effective [55,56,57]. The monkeypox infection cannot be cured with antibiotics; however, they can be used to control and bring to a halt secondary bacterial infections [46]. The smallpox vaccine offers cross-protection against MPXV [3]. In the fight against the smallpox virus, there have been three different generations of vaccinations used [58]. Up until 2008, the first-generation vaccination was the only one available for protection against smallpox [59,60]. This vaccination was extremely successful at preventing smallpox and played a crucial role in the elimination of the disease worldwide [60]. Some populations considered to be at a high risk of contracting orthopoxviruses have benefited from the use of a live attenuated vaccine (second-generation). A third-generation vaccine, the modified vaccinia Ankara-Bavarian Nordic (MVA-BN), is currently licensed for use in humans in both Europe and Canada [61,62]. 

Vaccines are crucial for eradicating infectious diseases like monkeypox [63]. Recent reports have indicated that in general, people are hesitant to be vaccinated in response to the current monkeypox outbreak [64,65,66,67,68,69,70,71,72]. There were numerous reasons cited for vaccine hesitancy, including the fear for negative side effects, misinformation, and mistrust of medical staff or the healthcare system [73,74]. Lack of knowledge and inappropriate attitudes of healthcare workers (HCWs) themselves might have a negative impact on their selection of treatment for themselves, and this might also affect the steps taken for primary prevention, early identification, and prompt intervention involving patients [75,76]. The lack of a comprehensive understanding of monkeypox among HCWs might be ascribed to several factors. Because monkeypox was traditionally thought to be confined to several areas in Africa, medical practitioners were not expecting to encounter it in their day-to-day practice. Second, it is challenging to diagnose monkeypox accurately because of the similarity of its symptoms with more frequently occurring diseases like chickenpox and smallpox [77]. Furthermore, the scarcity of reported monkeypox cases or the reporting of isolated cases had impeded the channeling of research funds and resources specific to this disease, hence impeding comprehensive investigations into its transmission, clinical presentations, and therapeutic interventions.

When a new infectious disease is discovered, it can be helpful to examine healthcare workers’ extent of knowledge, attitudes, and vaccine hesitancy. This fits with previous findings showing that misinformation about the dangers of an emerging infectious pathogen can increase anxiety, worry, and even the possibility of conspiratorial thinking [33,65,78,79]. In addition, bridging knowledge gaps might be considered a crucial component for the prevention of a monkeypox epidemic [80]. Healthcare workers’ knowledge, attitudes, and willingness to vaccinate against monkeypox are crucial to the development and implementation of an effective infection control strategy. To date, there have been relatively few studies carried out to ascertain the degree of knowledge and attitudes towards monkeypox among HCWs, college students, and the general public [39,59,60,64,65,81,82,83,84,85,86,87,88,89,90,91,92,93,94,95,96,97]. To the best of our knowledge, none of the published studies had assessed HCWs’ level of knowledge of monkeypox in Iraq. Since monkeypox infections can spread quickly and develop into an epidemic, more studies are required, particularly those concerning healthcare professionals. Healthcare professionals play a pivotal role in responding to a rising epidemic [98,99,100]. 

The Kurdistan region of Iraq has a population of around 6,000,000 and is in the Middle East. Although human monkeypox infections had not been recorded in the Kurdistan region of Iraq as of August 2023, the rapid growth of cases worldwide and the detection of monkeypox in neighboring Middle Eastern nations call for careful planning and timely response measures [35,101]. The worldwide monkeypox outbreak has had a substantial influence on healthcare systems and HCWs globally, including for those residing in the Kurdistan area. The situation led to an escalation in alertness and readiness within the healthcare sector, as additional resources and HCWs are allotted to assist with, monitor, and address potential cases, resulting in an increased demand for manpower and added workload. Taking into account the major role of healthcare workers in responding to the current emerging monkeypox epidemic [102,103], the present study aimed at assessing the knowledge, attitudes, and willingness of HCWs in the Kurdistan region of Iraq to vaccinate against monkeypox.

## 2. Materials and Methods

### 2.1. Study Design and Setting

The cross-sectional study was conducted among HCWs in the Kurdistan region of Iraq between 1 November 2022 and 15 January 2023. This study included Iraqi Kurdistan-registered healthcare workers. Those healthcare workers practiced in many cities across the region and have different medical specialties.

### 2.2. Sample Size and Participants

We determined the appropriate sample size by utilizing the Raosoft calculator. We established the size of the sample based on the most recent statistics, estimating that there were 30,000 HCWs working in the medical field across the Kurdistan region of Iraq in early 2022 [104]. Considering that there have been no prior studies in the Kurdistan region examining HCWs’ knowledge of monkeypox, a 50% conservative estimate was used. A minimum of 380 HCW samples were required to achieve a 5% margin of error with a 95% confidence interval. 

To recruit participants, convenience sampling was utilized. We derived a list of all HCW from the department of workforce. Google Forms was used to create the survey, and the participants were contacted via their respective heads of department at their respective healthcare centers. Each respondent was required to provide their informed consent by clicking on the agreement statement before starting to fill out the answers. A questionnaire was deemed complete and valid for inclusion when all the questions were filled. No rewards were awarded. Moreover, participation was totally on a voluntary basis. To ensure confidentiality, participants’ identities were not collected. The process of filling out the questionnaire required a total of ten minutes. Our target group comprised doctors, registered nurses, dentists, pharmacists, and medical technicians (MTs), aged 20 or older, who were willing to participate voluntarily and who were residing in the Kurdistan region of Iraq during the study period. None of the participants were excluded.

### 2.3. Study Tools

The researchers used a structured questionnaire with 34 items, including items requesting sociodemographic information. The research questionnaire was developed based on previously published studies addressing monkeypox knowledge, attitude, and willingness to vaccinate [71,72,86,88,91,105]. The questionnaire was drafted in English and Kurdish (the local language). The questionnaire was translated into the Kurdish language by different experts (virologists, epidemiologists). A forward-backwards translation from English to Kurdish ensured precise results. There were four main parts to the questionnaire, as outlined: (1) The first part included 8 items about sociodemographic data such as age, gender, marital status, highest educational level (undergraduate or postgraduate), duration of practice (in years), occupational category (doctor, dentist, pharmacist, nurse, or other medical technician), place of residence (inside the capital or outside the capital), and one “Yes” or “No” question about whether or not the respondent had heard about monkeypox before the year 2022. (2) The second part included 16 items related to knowledge on monkeypox transmission, prevention, treatment, and vaccination. (3) The third part included 5 items about attitudes towards monkeypox containment. (4) The fourth part included 5 items relating to the willingness to receive a monkeypox vaccination.

On 29 October 2022, a pilot study of 44 participants was conducted to assess the questionnaire’s reliability (the results were excluded from the final analysis). To determine whether the items had a consistent internal structure, Cronbach’s alpha was computed. Cronbach’s alpha was 0.77 for the knowledge scale, 0.74 for the attitude scale, and 0.75 for the willingness to vaccinate scale. The overall Cronbach’s alpha for the full questionnaire was 0.75, which demonstrated an appropriate level of internal consistency [106].

### 2.4. Study Variables 

There were 16 questions on the knowledge part, and the choices were “Correct”, “Incorrect,” and “Don’t Know”. A score of 1 represented a “correct” response, and 0 represented an “Incorrect” or “Don’t Know” response. Responses to inverted questions were assigned points in reverse (correct = 0 and incorrect = 1). Therefore, the overall score ranged from 0 to 16, with a higher score (12–16) indicating good knowledge and a score below 12 indicating poor knowledge about monkeypox disease.

The attitude part included 5 items, and the response to each item was measured on a 3-point Likert scale. A score of 1 represented “Agree”, and 0 represented “Undecided” or “Disagree”. The overall score ranged from 0 to 5, with a higher score (4–5) indicating a positive attitude and score below 4 indicating a negative attitude towards monkeypox disease.

The willingness to vaccinate against monkeypox part included 5 items, and the response to each item was measured on a 3-point Likert scale. A score of 1 represented “Agree”, and 0 represented “Undecided” or “Disagree”. The overall score ranged from 0–5, with a higher score (4–5) indicating that the respondent was “willing” to receive the monkeypox vaccine if it were made available, and a score below 4 indicated that the respondent was “unwilling” to receive the monkeypox vaccine. 

### 2.5. Ethical Consideration

The ethical approval was granted by the Research Ethics Committee of the University of Raparin, College of Basic Education, Department of Kindergarten, with a reference number 78/2023. This research followed the principles outlined in the Declaration of Helsinki. The confidentiality and anonymity of the participants’ responses were also guaranteed. The electronic informed consent form was obtained from all participants involved in the study. 

### 2.6. Data Analysis

All data analysis was performed using IBM SPSS Statistics version 25 (IBM Corporation, Armonk, NY, USA). Categorical variables and sociodemographic characteristics were presented as frequencies and percentages. Then, for determining the normality distribution of the data, the Shapiro–Wilk test was performed, and a histogram was plotted, and all the variables followed the normal distribution. The standard error of the skewness coefficient and the standard error of the kurtosis coefficient were also utilized to assess the normality of the data. Additionally, the chi-square test was used to determine the association between baseline sociodemographic characteristics and the scores for knowledge, attitude, and willingness to be vaccinated against monkeypox. The univariate binary logistic regression analysis was performed, and the variables with a *p*-value of ≤0.25 were selected for the multivariate binary logistic regression analysis. From the binary logistic regression, variables with a significance *p*-value of ≤0.05 and an odds ratio of 95% CI were considered as statistically significant variables that were independently associated with good knowledge, a positive attitude, and willingness to receive vaccination against monkeypox.

## 3. Results

### 3.1. Baseline Characteristics and Demographic Data

Of the 801 health workers invited, 637 responded to the survey (79.5% response rate) and whose data were used for analysis. The ages ranged between 21 and 51 years. Males represented 49.9% of participants, females represented 50.1%, and 49.3% of participants were single. A majority, 96.7%, received a diploma or a BSc degree. Of the 637 participants, 42.5% had 1 to 5 years of experience in delivering healthcare services. A third of the participants (35.6%) were nurses. Many of them, 73.9% of the participants, were from outside the capital city (Erbil). Finally, 60% of the participants reported that they had heard about monkeypox before the 2022 outbreak (Table 1)**.**

### 3.2. Knowledge, Attitude, and Willingness to Vaccinate against Monkeypox among HCWs

Table 2 shows that 74% of the participants answered the question that monkeypox is a viral disease correctly, and 69.5% answered the question accurately that monkeypox is not a bacterial infection. A majority, 68.3%, answered the question that monkeypox is transmitted from one person to another correctly, and 69.4% answered the question that monkeypox is transmitted to humans through direct contact from infected animals correctly. About 59% of the participants reported that monkeypox infection is associated with typical skin lesions, which is correct. Finally, 60.4% correctly identified that antivirals are required in the management of human monkeypox infections.

Table 3 demonstrates the distribution of the participants’ responses in frequencies and percentages. A total of 63.4% were interested in learning more about monkeypox. A total of 66.2% reported that the mass media coverage of monkeypox has influenced prevention globally, and 62.8% of the participants revealed they thought that mass media coverage of monkeypox may have an influence on its worldwide prevention. Finally, 72.8% of the participants said they thought that it is dangerous to travel to the countries in which monkeypox was an epidemic. For the variables of willingness to receive the vaccination for monkeypox, 62% of the participants reported that they were considering a smallpox vaccine to prevent against monkeypox infection, and 52.3% of the participants said that the recommendation for the vaccination by doctors, community pharmacists, and other professionals had a great influence on them.

**Figure 1:**
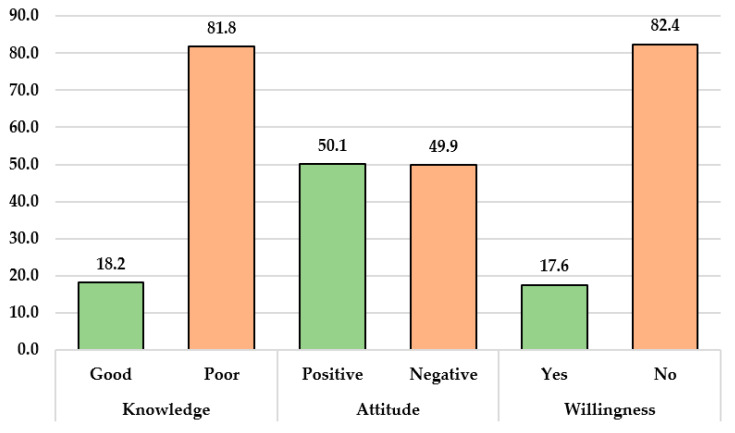
Distribution of knowledge (good and poor), attitudes (positive and negative), and willingness to be vaccinated (yes and no) by healthcare workers in the Kurdistan region, northern Iraq.

The mean scores and standard deviation for knowledge, attitude, and willingness were 8.18 ± 3.37, 3.40 ± 1.37, and 2.41 ± 1.25, respectively. Consequently, 116 participants (18.2%) had attained a good level of knowledge based on a 75% cutoff [107] (i.e., 12 questions answered correctly), and 319 participants (50.1%) demonstrated a positive attitude toward monkeypox. Nonetheless, only 112 participants (17.6%) were willing to receive the monkeypox vaccine if it were provided (Figure 1).

### 3.3. Relationships between Baseline Sociodemographic Characteristics and Knowledge, Attitude, and Willingness Scores regarding Monkeypox

Table 4 shows the association between participants’ sociodemographic characteristics and their knowledge, attitude toward monkeypox, and willingness to receive the vaccine against monkeypox. A good knowledge on the part of participants was associated with hearing about monkeypox before 2022 *(p* < 0.01). A positive attitude among participants was associated with age groups (*p* = 0.01), marital status (*p* < 0.01), duration of practice (*p* < 0.01), and place of residence (*p* = 0.03). High attitude scores were common among 31–41-year-old participants (56.1%), those more than 41 years old (66.9%), males (74%), those who were married (58.9%), and widow/er participants (63.2%). For undergraduates, both levels of attitude were equal (50.0%), whereas for the group of graduates, a positive attitude was common (52.4%). Also, high attitude scores were common among nurses (52.9%), physicians (54.3%), and residents of the capital city of “Erbil” (57.2%). Finally, the participants that heard about monkeypox before 2022 had high attitude scores (51.3%). The chi-square results showed that a willingness toward receiving the monkeypox vaccination was not associated with any of the baseline sociodemographic characteristics.

### 3.4. Univariate Binary Logistic Regression Analysis of Knowledge, Attitude, and Willingness toward Monkeypox Vaccination

Table 5 shows the univariate binary logistic regression analysis results and revealed that the only factor that was significantly associated with good knowledge about monkeypox was having heard about monkeypox before 2022 compared with participants who did not hear about monkeypox before 2022 (OR: 4.54; 95% CI: 2.67–7.73; *p* < 0.01). Also, the results showed that factors that were significantly associated with a positive attitude toward monkeypox were: 21–30-year-old participants compared to the participants aged 31–40 years old (OR:0.51; 95% CI: 0.29–0.89; *p* = 0.018); participants with less than 1 year of practice compared to the participants who had 1–5 years of practice (OR:27; 95% CI: 0.17–0.42; *p* < 0.01); and participants who lived outside the capital compared to the participants from the capital city of Erbil (OR: 0.67; 95% CI: 0.47–0.96; *p* = 0.033). Finally, none of the factors was significantly associated with the willingness to be vaccinated against monkeypox.

### 3.5. Multivariate Binary Logistic Regression Analysis of Knowledge, Attitude, and Willingness toward Monkeypox Vaccination

In the Table 6, the results of the multivariate binary logistic regression analysis showed that participants who had heard about monkeypox before 2022 were more likely to have good knowledge about monkeypox compared to the participants who did not hear about monkeypox before 2022 (AOR:4.85; 95% CI: 2.81–8.36; *p* < 0.001). Also, the results showed that participants with less than 1 year of practice were more likely to have a positive attitude towards monkeypox compared to participants who had 1–5 years of practice (AOR: 0.35; 95% CI: 0.20–0.59; *p* < 0.01). Finally, the results showed that none of the variable groups had the capacity to predict willingness to be vaccinated against monkeypox.

## 4. Discussion

This study represents the first attempt to assess the knowledge, attitudes, and willingness of healthcare workers (HCWs) in the Kurdistan region of Iraq with respect to human monkeypox and the associated vaccination. The factors that influenced their knowledge, attitudes, and willingness to be vaccinated were also explored.

The international healthcare system has faced one of the most significant challenges in recent decades with the outbreak of COVID-19. The pandemic not only overwhelmed healthcare systems worldwide but also put immense strain on healthcare workers, who were required to provide care for both non- and COVID-19 patients [108]. The recovery phase of the COVID-19 pandemic was overcome with hope when vaccination campaigns were initiated to control the spread of the virus. As the healthcare system was starting to regain its footing, the re-emergence of monkeypox presented a new challenge. The resurgence of monkeypox during the resolution phase of the COVID-19 pandemic has further strained the healthcare system, especially considering that many infection prevention measures implemented during the COVID-19 pandemic were gradually relaxed or abandoned. This unexpected development highlights the importance of maintaining a vigilant approach toward infectious disease prevention and control measures in healthcare settings [109]. Investigating healthcare workers’ knowledge and attitudes related to monkeypox also allows for targeted educational interventions. Identifying knowledge gaps or misconceptions can help in the development of tailored training programs to enhance HCWs’ understanding of the disease. By addressing these gaps, healthcare workers can improve their ability to recognize monkeypox cases accurately, provide appropriate care, and educate patients and the community about preventive measures [110].

Our study showed that 50.1% of HCWs were females, 49.3% of them were single, and 96.7% of the participants were undergraduates (diploma or BSc degree) with 1–5 years of experience.

Also, we found that 60% of HCWs had heard about monkeypox before the 2022 outbreak, which can be attributed to their medical education and training, engagement in professional development activities, and exposure to research and publications. These factors contributed to their overall awareness and knowledge of various infectious diseases. 

In this study, gaps in monkeypox knowledge were most conspicuous for issues of transmission from one person to another, how to differentiate between the clinical features of monkeypox and smallpox, specific vaccines, and treatment for monkeypox. Less than 40% of the HCWs correctly answered these questions. Overall, of HCWs from different categories who participated in this study, more than 80% had poor knowledge. Along the same line, recent studies that assessed HCWs’ knowledge in a Middle Eastern country [86] reported a low level of monkeypox knowledge among HCWs. On the other hand, a previous study conducted on HCWs in Saudi Arabia found that overall knowledge about monkeypox was relatively good [111]. Our study found that only 18.2% of HCWs had good knowledge about monkeypox, which is consistent with several prior studies. A survey of medical personnel in Italy showed that 53% of physicians scored inadequately on a 24-item monkeypox knowledge assessment [105]. Another study in Indonesia found that only about 10% of 432 general practitioners possessed knowledge of monkeypox exceeding 80% on a 21-item scale [90]. In a cross-sectional study in Bangladesh, out of 389 practicing medical physicians, only 30.59% demonstrated adequate knowledge of monkeypox [112]. In Jordan, among 606 health professionals (comprising roughly two-thirds physicians and nurses), only four out of eleven items related to monkeypox knowledge received correct responses at a 50% rate [86]. Additionally, among 651 medical college students, less than half were aware of the monkeypox outbreak [113].

The significance of this result shows that training HCWs in infectious diseases like monkeypox enables them to implement effective prevention and control measures, and HCWs trained in monkeypox are better equipped to recognize the signs and symptoms of the disease. Early detection is vital for initiating appropriate isolation measures and implementing supportive care promptly, as is differentiating monkeypox from other similar conditions, ensuring accurate diagnosis and appropriate management. Medical professionals in Muslim communities have unique obstacles when addressing a monkeypox outbreak because of religious restrictions to discussing certain aspects of patients’ sexual health. Additionally, aspects of males participating in sexual activities with other men are frowned upon, and these hamper contact-tracing operations. Healthcare workers are required to manage those sensitive issues while undertaking preventative measures.

In our data analysis, it was found that only 50% of HCWs displayed a positive attitude toward monkeypox, and 82.4% were not willing to be vaccinated against monkeypox. In line with our results, a cross-sectional study conducted across 27 countries indicated that 51.7% of participants exhibited a positive attitude towards monkeypox [114]. Conversely, in other studies conducted among healthcare workers in Saudi Arabia, the majority of participants displayed a positive attitude towards monkeypox [91,115]. The public vaccination efforts of HCWs are notably impacted by the positive attitudes they hold towards monkeypox vaccination. Public confidence is bolstered and vaccination rates are increased when HCWs demonstrate assurance in the safety and efficacy of the vaccine [116]. The community is reassured of the vaccine’s benefits, and misinformation is refuted through the influence of their potent endorsement. Elevated vaccination rates and improved public health outcomes result from the favorable atmosphere fostered by healthcare personnel who adopt a supportive stance. The attitude of HCWs plays a critical role in the management and control of monkeypox. HCWs with a positive attitude are more likely to be attentive and proactive in identifying potential cases of monkeypox and will also be effective communicators with patients and family [110]. The comparatively low positive attitudes regarding monkeypox vaccination among HCWs can be related to a lack of information and comprehension of the illness, its prevalence, and vaccine efficacy. Concerns about vaccination safety, possible adverse effects, and distrust in the healthcare system, along with misinformation and cultural or religious beliefs, all contribute to HCWs’ apprehension. Disparities in vaccination rates result from varying access to healthcare, education, and economic stability across different regions. The success of public health campaigns, immunization infrastructure, and political commitments also influence acceptance rates.

The finding of a monkeypox vaccine acceptance rate of only 17.6% in the study is significantly lower compared to the pooled estimate from a recent meta-analysis. This meta-analysis involved HCWs from four different studies, which reported an acceptance rate of 63% [117]. The difference in acceptance rates between this study and the meta-analysis raises important considerations regarding monkeypox vaccination hesitancy among health professionals. It is worth noting that the acceptance rate reported among HCWs in the meta-analysis is higher than the rates reported among the general public worldwide. Lounis and Riad conducted a recent review that highlighted the issue of possible monkeypox vaccination hesitancy among health professionals despite their relatively higher rates of vaccine acceptance compared to the general public [118]. Furthermore, another recent study among medical workers in China described a majority of participants supporting the promotion of monkeypox vaccination, mainly among health practitioners and immune-deficient populations [119]. A study conducted on adults in the United States revealed that knowledge levels regarding monkeypox vaccination were low, and only 46% expressed intentions to be vaccinated if the vaccine was recommended. This suggests a lack of awareness and understanding about the importance and benefits of monkeypox vaccination [120].

In contrast, a recent study conducted among Pakistani university students aimed to assess their knowledge, attitudes, and willingness to be vaccinated against monkeypox. The findings of the study revealed that there is a relatively high level of willingness among the students to receive monkeypox vaccination, with 68% expressing their willingness. Additionally, 35% of the participants were willing to pay for the vaccine [121]. An additional study among HCWs in China showed high willingness to receive the monkeypox vaccination, at a rate of 90%.

Vaccine hesitancy refers to a delay in the acceptance of or the refusal of vaccines, regardless of their availability. It frequently emerges because of apprehensions over safety, inadequate knowledge, and a lack of confidence. To effectively tackle this issue, it is imperative to ensure the provision of precise and reliable information, foster open dialogues, and actively include HCWs in the decision-making process. These measures are crucial in fostering trust among individuals and communities. Customized educational initiatives and practical illustrations of vaccination in real-world scenarios have the potential to debunk misconceptions and enhance public trust in vaccines. Vaccine hesitancy is an intricate phenomenon influenced by various factors such as individual beliefs, attitudes, knowledge, and social influences. Thoughtful vaccine hesitancy among HCWs is crucial, as they play a dynamic role in promoting vaccination and ensuring public health. The organizational aspects within healthcare settings that can affect vaccine acceptance among HCWs, such as vaccine availability, accessibility, and convenience, can impact their willingness to be vaccinated. Financial barriers can also be addressed by exploring options for making the vaccine more accessible and affordable. This could involve negotiating with vaccine manufacturers for reduced prices or seeking funding from the government or non-governmental organizations to subsidize the cost of vaccination. 

Complacency towards monkeypox vaccination may arise due to a perception that the disease is not a significant threat in certain regions. Another factor contributing to vaccine reluctance could be misinformation or misconceptions about monkeypox and its vaccine. Misinformation can spread through various channels, including social media platforms, websites, and word of mouth [119].

Furthermore, cultural and religious beliefs can also play a role in vaccine reluctance. Some individuals may have concerns about the use of vaccines due to religious or cultural beliefs that discourage medical interventions. These beliefs can vary across different communities and may influence an individual’s decision regarding vaccination [107]. 

Additionally, mistrust in healthcare systems or government authorities can contribute to vaccine hesitancy. If individuals have doubts about the transparency or effectiveness of vaccination campaigns, they may be less likely to trust and comply with recommended immunization practices. Building trust through transparent communication, providing accurate information, and addressing concerns can help mitigate vaccine hesitancy [111]. To combat public vaccine hesitancy and increase rates of monkeypox immunization, HCWs must play a pivotal role. HCWs can encourage trust in vaccination by setting a good example and getting vaccinated themselves, as well as by offering their professional advice to others. They can interact with patients and communities to clarify misinformation and answer concerns about the monkeypox vaccination. HCWs may use their credibility to promote vaccination through events, campaigns, and online forums. Furthermore, they can work together with community leaders, religious figures, and officials to advocate for vaccination, stressing the significance of stopping the spread of monkeypox. It is crucial to keep in mind that vaccine reluctance is a complex issue that depends on a variety of factors, some of which can vary across populations and regions. Understanding the specific reasons behind vaccine hesitancy in a particular context is crucial for designing targeted interventions to address these concerns effectively [122].

In this study, we found that good knowledge of HCWs regarding monkeypox was associated with hearing about monkeypox before 2022. This suggests that individuals who had prior awareness of monkeypox were more likely to possess a higher level of knowledge regarding the disease. 

Additionally, a positive attitude towards monkeypox was linked with HCWs who were aged between 31 and 41 years old; this group showed an even higher percentage (66.9%) of positive attitudes. Marital status was also found to be significantly associated with a positive attitude (*p* < 0.01). Furthermore, HCWs with longer durations of practice were more likely to have positive attitudes towards monkeypox. 

When considering educational background, the study found that among under undergraduates, both levels of attitude (positive and negative) were equal, with 50% exhibiting high attitude scores. However, among the graduate group, a higher percentage displayed positive attitudes, at 52.4%. In terms of professional backgrounds, nurses had a relatively high prevalence of positive attitudes, with 52.9% scoring high on attitude measures. Physicians also showed a significant proportion of positive attitudes at 54.3%. Lastly, we found that geographically, the capital city (Erbil) had a relatively higher percentage of participants with positive attitudes at 57.2%.

In the current survey, a multivariate logistic regression analysis was conducted to identify additional determinants that have a significant influence on the different outcomes of interest. Specifically, the analysis revealed that the number of years of working activity in the healthcare profession and gender were predictive factors for knowledge and attitude among the sampled healthcare workers (HCWs). One possible explanation for less experienced HCWs being more active in acquiring information, reading scientific journals, and participating in recent proper training and education compared to those with more years of activity is their higher level of enthusiasm and motivation. Additionally, less experienced HCWs may have a stronger desire to establish themselves professionally and build a solid foundation of knowledge and skills. Furthermore, younger HCWs who are just starting their careers may be more comfortable with technology and digital platforms, which can facilitate access to scientific journals, online courses, webinars, and other educational resources. They may be more adept at utilizing these tools for self-directed learning compared to their more experienced counterparts, who may have been trained in a different era.

Attitudes and willingness to be vaccinated can vary based on the type of vaccine and the population of interest. This study emphasizes the need for special attention to the intricate details surrounding this topic. It acknowledges that previous evidence suggests that vaccine mandates may not always be effective due to divided opinions among the general public and healthcare workers (HCWs), the latter of whom can play a crucial role in advocating for such strategies [123,124].

## 5. Strengths and Limitations

A major strength of this study lies in its ability to collect data from a representative sample of different hospitals inside the Kurdistan region, to provide an overview of knowledge gaps, and to display attitudes and monkeypox vaccine willingness among HCWs. These gaps and attitudes can significantly impact the effectiveness of public health interventions and the overall control of the outbreak.

This study’s limitations included, firstly, the cross-sectional design of the survey, which is a limitation. A cross-sectional study collects data at a single point in time, providing a snapshot of a population or phenomenon. Though this design allows for the examination of associations between variables, it does not establish causality. Additionally, one potential limitation of using self-administered questionnaires is that participants may feel pressured to present themselves in a positive light or conform to societal norms. This can result in respondents providing answers that they believe are more socially acceptable rather than reflecting their true thoughts, feelings, or behaviors.

## 6. Conclusions

A gap in the level of knowledge of monkeypox was evident among HCWs. One of the primary challenges in identifying and managing monkeypox cases is the lack of awareness among healthcare professionals and the public. Without adequate understanding and awareness of the signs, symptoms, and the mode of transmission of monkeypox, it is difficult to recognize and respond to potential cases effectively. This can lead to a delay in diagnosis, early treatment, and containment measures. It is imperative that the implementation of timely campaigns, educational courses, conduct of seminars and workshops succeed so that HCWs are equipped with a better understanding of monkeypox, hence enhancing their ability to provide high-quality and safe patient care. To promote vaccine acceptance, reliable information, inclusive debates, and honest communication will be helpful. The sharing of success stories of vaccination and a supportive workplace can boost healthcare professionals’ inclination to vaccinate. One of the key actions that can be taken to combat vaccine hesitancy is enhancing education and awareness regarding the vaccine’s components, effects, and importance for both HCWs and the general population. Furthermore, it is essential to establish vigorous surveillance systems that enable early detection of monkeypox cases. These systems should involve close collaboration between healthcare facilities, laboratories, public health agencies, and other relevant stakeholders. By implementing real-time monitoring and reporting mechanisms, potential monkeypox cases can be identified promptly, allowing for timely intervention and containment measures.

Finally, social media platforms could collaborate with public health organizations to ensure that accurate and up-to-date information is readily available to users. This can be effectuated through partnerships that involve sharing verified information, promoting official healthcare-related guidelines, and providing resources for users.

## Figures and Tables

**Table 1:** Sociodemographic characteristics of healthcare workers (n = 637).

Variables	Items	Frequency	Percentage
Age	21–30	401	63.0
	31–40	173	27.2
	41+	63	9.8
Gender	Male	318	49.9
	Female	319	50.1
Marital status	Single	314	49.3
	Married	304	47.7
	Widow/er	19	3
Level of education	Undergraduate (diploma or BSc degree)	616	96.7
	Postgraduate (MSc, PhD, or its equivalent)	21	3.3
Work experience	Less than 1 year	168	26.4
	1–5 years	271	42.5
	More than 5 years	198	31.1
Occupational category	Medical technicians	130	20.4
	Nurse	227	35.6
	Pharmacist	121	19.0
	Dentist	65	10.2
	Physician	94	14.8
Residence area	The capital (Erbil)	166	26.1
	Outside the capital	471	73.9
Heard about the human monkeypox before 2022?		Yes	No
		382 (60%)	255 (40%)

**Table 2:** Knowledge of healthcare workers towards monkeypox (n = 637).

Knowledge Items	Correct Answers n (%)	Incorrect Answers n (%)
Q1. Monkeypox is a new infection that appeared this year, 2022.	324 (50.9)	313 (49.1)
Q2. Monkeypox is a viral disease infection.	476 (74.7)	161 (25.3)
Q3. Monkeypox is a bacterial infection.	443 (69.5)	193 (30.3)
Q4. Monkeypox is prevalent in Middle Eastern countries.	283 (44.4)	354 (55.6)
Q5. Monkeypox is prevalent in Western and Central Africa.	334 (52.4)	303 (47.6)
Q6. There are many human monkeypox cases in Iraq.	328 (51.5)	309 (48.5)
Q7. Monkeypox is transmitted from one person to another.	435 (68.3)	202 (31.7)
Q8. Monkeypox is transmitted to humans through direct contact from infected animals.	442 (69.4)	195 (30.6)
Q9. Monkeypox is spread by droplets (coughing and sneezing).	320 (50.2)	317 (49.8)
Q10. Monkeypox and smallpox have similar signs and symptoms.	176 (27.6)	461 (72.4)
Q11. Monkeypox infection is associated with typical skin lesions.	377 (59.2)	260 (40.8)
Q12. Lymphadenopathy (swollen lymph nodes) is one clinical sign or symptom that could be used to differentiate between monkeypox and smallpox cases.	275 (43.2)	362 (56.8)
Q13. There is a specific vaccine for monkeypox.	195 (30.6)	442 (69.4)
Q14. There is a smallpox vaccine that can be used for monkeypox.	197 (30.9)	440 (69.1)
Q15. There is a specific treatment for monkeypox.	224 (35.2)	413 (64.8)
Q16. Antivirals are required in the management of human monkeypox patients.	385 (60.4)	252 (39.6)

**Table 3:** HCWs’ attitude towards monkeypox and willingness to vaccinate (n = 637).

	Question No.	Questions	Agree No. (%)	Disagree or Undecided No. (%)
Attitude toward monkeypox				
	A1	I am confident that the spread of monkeypox infection can be controlled worldwide.	404 (63.4)	233 (36.6)
	A2	I am interested in learning more about monkeypox.	484 (76)	153 (24.0)
	A3	I think that monkeypox can add a burden on healthcare systems of affected countries.	422 (66.2)	215 (33.8)
	A4	I think that mass media coverage of monkeypox influenced prevention worldwide.	400 (62.8)	237 (37.2)
	A5	I think that it is dangerous to travel to the countries where monkeypox cases were detected.	460 (72.2)	177 (27.8)
Willingness of HCWs to be vaccinated against monkeypox				
	W1	I am considering getting the smallpox vaccine to prevent against contracting monkeypox infection.	395 (62)	242 (38.0)
	W2	The monkeypox infection has been alleviated, and there is no need to be vaccinated against the monkeypox virus.	177 (27.8)	460 (72.2)
	W3	I am worried about the possible side effects of the monkeypox vaccine.	307 (48.2)	330 (58.1)
	W4	The recommendation for the vaccination by doctors, community pharmacists, and other healthcare professionals has had a great influence on me.	333 (52.3)	188 (29.5)
	W5	If the country provides a vaccination against monkeypox infection for free, I am willing to be vaccinated.	327 (51.3)	304 (47.7)

**Table 4:** Association between baseline sociodemographic characteristics and knowledge, attitude, and willingness scores regarding monkeypox (n = 637).

Variables		Knowledge				Attitude				Willingness			
		Good	Poor	X^2^	*p*-Value	Positive	Negative	X^2^	*p*-Value	Yes	No	X^2^	*p*-Value
Age (years)	21–30	73 (18.2)	328 (81.8)	0.03	0.98	183 (45.6)	218 (54.4)	9.17	0.01	63 (15.7)	338 (84.3)	2.61	0.27
	31–40	32 (18.5)	141 (81.5)			97 (56.1)	76 (43.9)			36 (20.8)	137 (79.2)		
	41+	11 (17.5)	52 (82.5)			39 (61.9)	24 (38.1)			13 (20.6)	50 (79.4)		
Gender	Male	54 (16.9)	265 (83.1)	0.70	0.40	162 (74.0)	157 (26.0)	0.12	0.72	50 (15.7)	269 (84.3)	1.60	0.20
	Female	62 (19.5)	256 (80.5)			157 (49.4)	161 (50.6)			62 (19.5)	256 (80.5)		
Marital status	Single	55 (17.5)	259 (82.5)	2.71	0.25	128 (40.8)	186 (59.2)	21.6	<0.01	57 (18.2)	257 (81.8)	0.16	0.92
	Married	60 (19.7)	244 (80.3)			179 (58.9)	125 (41.1)			52 (17.1)	252 (82.9)		
	Widow/er	1 (5.3)	18 (94.7)			12 (63.2)	7 (36.8)			3 (15.8)	16 (84.2)		
Level of education	Undergraduate	115 (18.7)	501 (81.3)	2.63	0.10	308 (50.0)	308 (50.0)	0.04	0.83	109 (17.7)	507 (82.3)	0.16	0.68
	Graduate	1 (4.8)	20 (95.2)			11 (52.4)	10 (47.6)			3 (14.3)	18 (85.7)		
Duration of practice	Less than 1 year	31 (18.5)	137 (81.5)	1.54	0.46	52 (31.0)	116 (69.0)	37.0	<0.01	31 (18.5)	137 (81.5)	0.31	0.85
	1–5 years	44 (16.2)	227 (83.8)			144 (53.1)	127 (46.9)			45 (16.6)	226 (83.4)		
	More than 5 years	41 (20.7)	157 (79.3)			123 (62.1)	75 (37.9)			36 (18.2)	162 (81.8)		
Occupation	Medical technician	18 (13.8)	112 (86.2)	5.77	0.21	57 (43.8)	73 (56.2)	3.48	0.48	14 (10.8)	116 (89.2)	7.63	0.10
	Nurse	44 (19.4)	183 (80.6)			120 (52.9)	107 (47.1)			42 (18.5)	185 (81.5)		
	Pharmacist	29 (24.0)	92 (76.0)			59 (48.8)	62 (51.2)			22 (18.2)	99 (81.8)		
	Dentist	12 (18.5)	53 (81.5)			32 (49.2)	33 (50.8)			17 (26.2)	48 (73.8)		
	Physician	13 (13.8)	81 (86.2)			51 (54.3)	43 (45.7)			17 (18.1)	77 (81.9)		
Place of residence	Outside the capital	91 (19.3)	380 (80.7)	1.49	0.21	224 (47.6)	247 (52.4)	4.59	0.03	86 (18.3)	385 (81.7)	0.57	0.45
	Inside the capital (Erbil)	25 (15.1)	141 (84.9)			95 (57.2)	71 (42.8)			26 (15.7)	140 (84.3)		
Heard about human monkeypox before 2022	Yes	98 (25.7)	284 (74.3)	35.5	<0.01	196 (51.3)	186 (48.7)	0.57	0.44	65 (17.0)	317 (83.0)	0.21	0.64
	No	18 (7.1)	237 (92.9)			123 (48.2)	132 (51.8)			47 (18.4)	208 (81.6)		

X^2^ = chi-square.

**Table 5:** Univariate binary logistic regression analysis to evaluate the predictive factors associated high knowledge, attitude, and willingness scores regarding monkeypox (n = 637).

Items	Knowledge				Attitude				Willingness			
	OR	95% CI		P	OR	95% CI		P	OR	95% CI		P
Age (41+ as ref.)												
21–30 years	1.05	0.52	2.11	0.887	0.51	0.29	0.89	0.018	0.71	0.36	1.39	0.328
31–40 years	1.07	0.50	2.28	0.855	0.78	0.43	1.41	0.423	1.01	0.49	2.06	0.977
Gender (female as ref.)												
Male	0.84	0.56	1.25	0.401	1.05	0.77	1.44	0.721	0.76	0.50	1.15	0.206
Marital status (widow/er as ref.)												
Single	3.82	0.50	29.23	0.196	0.40	0.15	1.04	0.062	1.18	0.33	4.19	0.795
Married	4.42	0.57	33.81	0.152	0.83	0.32	2.18	0.713	1.10	0.30	3.91	0.882
Educational level (postgraduate as ref.)												
Undergraduate	0.21	0.02	1.64	0.139	0.90	0.38	2.17	0.830	1.29	0.37	4.45	0.687
Duration of practice (more than 5 years as ref.)												
Less than 1 year	0.86	0.51	1.45	0.589	0.27	0.17	0.42	< 0.01	1.01	0.59	1.73	0.947
Between 1 and 5 years	0.74	0.46	1.18	0.215	0.69	0.47	1.00	0.053	0.89	0.55	1.45	0.656
Occupation (physician as ref.)												
Medical technician	1.00	0.46	2.16	0.997	0.65	0.38	1.12	0.125	0.54	0.25	1.17	0.121
Nurse	1.49	0.76	2.93	0.238	0.94	0.58	1.53	0.820	1.02	0.55	1.91	0.930
Pharmacist	1.96	0.95	4.03	0.066	0.80	0.46	1.37	0.424	1.00	0.50	2.02	0.985
Dentist	1.41	0.59	3.32	0.432	0.81	0.43	1.54	0.533	1.60	0.74	3.44	0.225
Residence (inside the capital of “Erbil” as ref.)												
Outside the capital	1.35	0.83	2.18	0.223	0.67	0.47	0.96	0.033	1.20	0.74	1.94	0.450
Heard about monkeypox before 2022 (no as ref.)												
Yes	4.54	2.67	7.73	< 0.01	1.13	0.82	1.55	0.447	0.90	0.60	1.37	0.64

OR = odds ratio, CI = confidence interval, P = *p*-value.

**Table 6:** Multivariate binary logistic regression analysis to evaluate the predictive factors associated high knowledge, attitude, and willingness scores regarding monkeypox (n = 637).

Items	Knowledge				Attitude				Willingness			
	AOR	95% CI		P	AOR	95% CI		P	AOR	95% CI		P
Age (41+ as ref.)												
21–30 years	-	-	-	-	0.95	0.51	1.77	0.887	-	-	-	-
31–40 years	-	-	-	-	0.91	0.49	1.68	0.778	-	-	-	-
Gender (female as ref.)												
Male	-	-	-	-	-	-	-	-	0.78	0.51	1.20	0.268
Marital status (widow/er as ref.)												
Single	2.99	0.37	23.90	0.302	0.48	0.17	1.32	0.158	-	-	-	-
Married	3.42	0.42	27.37	0.246	0.70	0.26	1.88	0.481	-	-	-	-
Educational level (postgraduate as ref.)												
Undergraduate	4.45	0.56	35.15	0.156	-	-	-	-	-	-	-	-
Duration of practice (more than 5 years as ref.)												
Less than 1 year	0.95	0.50	1.79	0.875	0.35	0.20	0.59	< 0.01	-	-	-	-
Between 1 and 5 years	0.75	0.44	1.28	0.299	0.79	0.51	1.22	0.294	-	-	-	-
Occupation (physician as ref.)												
Medical technician	1.19	0.53	2.68	0.664	0.74	0.42	1.31	0.314	0.56	0.26	1.20	0.139
Nurse	1.82	0.89	3.72	0.099	1.07	0.64	1.79	0.787	1.03	0.55	1.93	0.916
Pharmacist	1.81	0.85	3.86	0.122	0.83	0.47	1.46	0.529	0.96	0.47	1.94	0.918
Dentist	1.51	0.62	3.70	0.360	0.83	0.42	1.61	0.586	1.62	0.75	3.47	0.215
Residence (inside the capital of “Erbil” as ref.)												
Outside the capital	1.38	0.82	2.31	0.215	0.81	0.55	1.19	0.294	-	-	-	-
Heard about monkeypox before 2022 (no as ref.)												
Yes	4.85	2.81	8.36	<0.001	-	-	-	-	-	-	-	-

AOR: adjusted odds ratio; ref.: reference; CI: confidence interval, P = *p*-value.

## Data Availability

Data are available from the first author upon reasonable request.
